# Assessing childhood maltreatment and mental health correlates of disordered eating profiles in a nationally representative sample of English females

**DOI:** 10.1007/s00127-015-1154-7

**Published:** 2015-11-25

**Authors:** Cherie Armour, Jana Műllerová, Shelley Fletcher, Susan Lagdon, Carol Rhonda Burns, Martin Robinson, Jake Robinson

**Affiliations:** School of Psychology, University of Ulster, Coleraine, Northern Ireland UK

**Keywords:** Eating disorders, Childhood trauma, PTSD, Depression, Suicide

## Abstract

**Purpose:**

Previous research suggests that childhood maltreatment is associated with the onset of eating disorders (ED). In turn, EDs are associated with alternative psychopathologies such as depression and posttraumatic stress disorder (PTSD), and with suicidality. Moreover, it has been reported that various ED profiles may exist. The aim of the current study was to examine the profiles of disordered eating and the associations of these with childhood maltreatment and with mental health psychopathology.

**Methods:**

The current study utilised a representative sample of English females (*N* = 4206) and assessed for the presence of disordered eating profiles using Latent Class Analysis. Multinomial logistic regression was implemented to examine the associations of childhood sexual and physical abuse with the disordered eating profiles and the associations of these with PTSD, depression and suicidality.

**Results:**

Results supported those of previous findings in that we found five latent classes of which three were regarded as disordered eating classes. Significant relationships were found between these and measures of childhood trauma and mental health outcomes.

**Conclusions:**

Childhood sexual and physical abuse increased the likelihood of membership in disordered eating classes and these in turn increased the likelihood of adverse mental health and suicidal outcomes.

## Introduction

An eating disorder (ED) is generally defined as a psychological illness which results in atypical eating behaviours such as insufficient or excessive eating habits [1]. EDs tend to be diagnosed under a number of distinct categories corresponding to the behavioural characteristics of specific symptoms [1]. The most well known of these is anorexia nervosa (AN), characterised by an extreme food intake restriction and an abnormal obsession to control and maintain low body weight [1]. Next is bulimia nervosa (BN) which can be characterised by large intakes of food in short periods of time (binging) followed by compensatory weight loss behaviours such as purging [1]. Both AN and BN are similar in that individuals strive to control body weight, but AN typically results in very low body weight whereas BN maintains an average body weight. Another ED is the binge eating disorder (BED) characterised by overeating with an absence of purging behaviours typically resulting in excess weight gain or obesity [1]. Additional categories of EDs included in the fifth edition of the Diagnostic and Statistical Manual of Mental Disorders (DSM-5) [2] include ‘other specified feeding or eating disorder’ (OSFED) and ‘unspecified feeding or eating disorder’ (UFED). OSFED/UFED are eating disorders of clinical significance but do not meet the diagnostic criteria for AN, BN or BED [3]. The elusive nature of the latter ED categories and the fact that ED patients have been noted as presenting variability in ED symptoms and diagnostic crossover within distinct categorical groups [4] have led researchers to consider the importance of symptom dimensions of EDs such as severity of symptoms within and across categories.

Within the UK, it is suggested that incidence rates of EDs have increased significantly over the past 10 years [5]. The Health and Social Care Information Centre (HSCIC) [6] for England found that in the 12 months prior to October 2013, hospital admissions for EDs had risen by 8 %. In total the HSCIC reported dealing with more than 2500 cases, 91 % of which were females. This is not surprising given the large body of literature which demonstrates a greater risk of ED development in females [7]. The HSCIC [6] also highlighted that the average age of admission for females was 15 years. Such reports reflect supporting literature suggesting that ED symptoms typically develop during adolescence with average diagnoses occurring between 15 and 19 years [7]. Symptoms associated with ED are chronic after onset, often continuing into adulthood [8]. Although treatment has been found to be effective in early diagnostic cases, residual symptoms such as body shape dissatisfaction and over concern with weight can continue [7]. It is suggested that the different types of EDs can also result in a variation of course and duration of the symptoms. For example, AN is noted as having longer duration and recovery periods compared to BN and BED [7]. Individuals suffering from AN are also more likely to remain chronically underweight, experience more physical health deficits, and are at increased risk of death due to starvation [8]. The HSCIC [6] reported that AN accounted for three in four hospital admissions for ED.

The aetiology of EDs is multi-factorial; however, there is compelling body of research suggesting that stressful life events are implicated in the onset and maintenance of EDs. Lejonclou, Nilsson and Holmqvist [9] compared a sample of ED patients (*n* = 50) with a non-clinical sample (*n* = 245) to assess the extent to which both groups had ever experienced a childhood and/or an adult trauma. Results revealed that ED patients had experienced a higher frequency of different traumas, in particular, childhood adversities. Such findings are consistently reported in the extant literature, especially in relation to childhood sexual abuse (CSA) [10]. Research suggests that CSA may be a strong predictor of the development of ED behaviours such as bulimic or binge eating symptoms [9].

Less research has addressed the relationship between EDs and other types of childhood adversities such as physical abuse. Kent and Waller [11] suggested that differing typologies associated with childhood adversities may contribute to the onset of particular ED pathologies. For example, childhood physical abuse (CPA) and peer bullying have been found to be associated with BED in females from diverse ethnic groups [12]. That being said, it was also reported that CPA and peer bullying were associated with the development of mental health disorders rather than representing a unique risk for the development of BED [12].

EDs have been commonly found to co-occur with additional mental health disorders, such as anxiety, depression, and post-traumatic stress disorder (PTSD) [13, 14]. For example, using data from the National Comorbidity Survey-Replication, Mitchell et al. [14] assessed the relationship between trauma history, EDs, and PTSD and found that females with a trauma history were more likely to also experience comorbid PTSD, BN, and BED.

Many symptoms associated with PTSD and EDs tend to overlap. Examples include emotional dysregulation and dissociation [14]. Behaviours, such as purging, may be a manifestation of dissociation whereby the individual is trying to avoid or regulate PTSD symptoms, further maintaining atypical eating behaviours through maladaptive coping [14]. Furthermore, the psychological impact associated with experiencing a childhood trauma is said to persist long into adulthood. On average, a diagnosis of PTSD or depression occurs during early to mid-adulthood [15]. It may, therefore, be the case that symptoms of PTSD are present in the form of preceding ED symptoms. If this is the case, specific treatment or intervention for the primary presenting disorder may not be effective due to later diagnosis of secondary disorders [15]. This may in turn result in increased risk of disorder severity and lack of treatment response. Such cumulative risk associated with the co-occurrence of secondary disorders has been found to impact on the risk of suicidal ideation in ED patients [16]. Further research incorporating dimensions of psychopathology are needed to help clarify within and across group differences.

The current study employed latent class analysis (LCA) to identify groups of adult females with specific patterns of eating behaviours such as those associated with previously discussed categories of EDs. Representative population data from the Adult Psychiatric Morbidity Survey (APMS) for England [17] was used. Subsequently, the association between experiences of CSA and CPA with each of the latent classes and the association of the classes with secondary mental health disorders were explored whilst controlling for a number of socio-demographic variables. The current research focused on a female-only sample given the vast body of literature which supports greater risk of EDs and associated outcomes in females [7, 14]. We hypothesised (1) that we will uncover a number of disordered eating profiles; (2) that females reporting CSA or CPA will be more likely to be grouped into disordered eating profiles compared to non-disordered eating profiles; and (3) that females grouped into disordered eating profiles will have an increased likelihood of reporting adverse mental health and suicidal outcomes.

## Methods

### Participants

The current study used secondary data from the APMS 2007 [17]. The APMS 2007 was the third in a series of surveys assessing the mental health of individuals aged 16 or over living in private households throughout England. Data were collected by the National Centre for Social Research in collaboration with the University of Leicester between October 2006 and December 2007. The final APMS sample consisted of 7403 usable cases (*n* = 4206 females). Sampling procedures have been described elsewhere [13].

### Measures

#### Socio-demographic characteristics

The following socio-demographic characteristics were included in the analyses: age, education, ethnicity, and marital status. These variables were dummy-coded, with the last category serving as the reference group (see Table 1).

**Table 1:** Descriptive statistics and childhood trauma experiences for the effective sample and individual classes

	Total sample	Class 1 (normal weight non-symptomatic)	Class 2 (obese non-symptomatic)	Class 3 (morbidly obese)	Class 4 (normal weight symptomatic)	Class 5 (obese binge eating)
	*N* (%)	*N* (%)	*N* (%)	*N (*%)	*N* (%)	*N* (%)
Participants	3845	2851 (73.6) *Wgt* 2832	588 (14.8) *Wgt* 570	95 (2.5) *Wgt* 94	144 (4.6) *Wgt* 179	167 (4.4) *Wgt* 170
Age						
16–34	807 (28.7)	593 (29.2)	77 (16)	11 (14.6)	69 (59)	57 (39.4)
35–54	1333 (35.4)	948 (33.8)	193 (37.2)	52 (57)	60 (33.2)	80 (46.5)
55+	1705 (35.9)	1310 (37.1)	318 (46.7)	32 (28.4)	15 (7.8)	30 (14.1)
Marital status						
Single	669 (20.3)	487 (20.5)	72 (11.7)	17 (14.9)	55 (46.5)	38 (22.3)
Previously married/cohabiting	1178 (19.4)	876 (19)	201 (24.3)	28 (21.4)	28 (9.7)	45 (18.6)
Married/cohabiting	1998 (60.2)	1488 (60.5)	315 (64.1)	50 (63.7)	61 (43.8)	84 (59.1)
Ethnicity						
White	3582 (91.3)	2666 (91.7)	547 (90.7)	85 (87.1)	132 (91.6)	152 (89.1)
Black	99 (3.1)	59 (2.4)	26 (6)	5 (5.2)	3 (2.5)	6 (4.3)
Asian	78 (2.7)	62 (3)	6 (1.4)	1 (1.2)	4 (2.6)	5 (4.1)
Mixed/other	80 (2.7)	60 (2.8)	8 (1.7)	4 (6.5)	4 (2.7)	4 (2.5)
Missing	6 (0.2)	4 (0.2)	1 (0.1)	–	1 (0.6)	–
Education						
No qualification	1273 (28.5)	949 (28.5)	235 (36.2)	38 (38.8)	20 (10.4)	31 (15.9)
Foreign/other	136 (2.9)	105 (3.1)	24 (3.3)	2 (3)	2 (0.9)	3 (1.5)
GCSE	977 (27.6)	683 (26.2)	154 (28.9)	32 (34.2)	54 (37.9)	54 (32.4)
A level	428 (12.8)	300 (12.1)	59 (11.2)	13 (12.7)	30 (23.2)	26 (17.8)
Higher level	973 (26.7)	777 (28.9)	102 (17.6)	9 (10.7)	36 (26.5)	49 (29.9)
Missing	58 (1.5)	37 (1.2)	14 (2.7)	1 (0.7)	2 (1.2)	4 (2.5)
Depressive episode	147 (3.4)	80 (2.2)	20 (3.3)	11 (11.6)	12 (9.2)	24 (12.2)
Probable PTSD	127 (3.1)	75 (2.3)	16 (2.6)	7 (8.4)	15 (10.2)	14 (8.6)
Missing	58 (1.4)	44 (1.3)	8 (1.4)	2 (2)	3 (1.6)	1 (0.6)
Attempted suicide	240 (5.7)	133 (4.2)	39 (6.3)	(12.1)	25 (14.2)	31 (16.1)
Missing	2 (0.1)	1 (0.1)	–	–	1 (1.3)	–
Physical abuse	164 (4.1)	81 (2.7)	31 (5.6)	13 (13.4)	13 (6.8)	26 (14.6)
Missing	2 (0.1)	2 (0.1)	–	–	–	–
Sexual abuse	662 (17.5)	418 (14.9)	96 (16.3)	35 (36.7)	53 (40.2)	60 (31.6)
Missing	21 (0.6)	15 (0.6)	3 (0.5)	–	2 (0.9)	1 (1.4)

% weighted, *n* unweighted, *Wgt* weighted class frequencies

#### Childhood trauma

Questions relating to CSA and CPA from the ‘violence and abuse’ section of the survey were included in the analyses. Respondents were asked the following:‘Before the age of 16 did anyone talk to you in a sexual way that made you feel uncomfortable?’; ‘Before the age of 16 did anyone touch you, or get you to touch them in a sexual way without your consent?’ and; ‘Before the age of 16, did anyone have sexual intercourse with you without your consent?’ Positive endorsement of one or more of the above questions classed the individual as having experienced CSA. One item in which the respondent was asked ‘Before the age of 16 were you severely beaten by a parent, step-parent or carer?’ was used to assess the CPA.

#### Eating disorders

Symptoms of EDs were assessed with the SCOFF questionnaire which is a dichotomous five item screening tool used to examine the possible presence of EDs. A score of two or more indicates possible presence of disordered eating [18]. The SCOFF questionnaire was administered as part of the self-completion section of Phase 1 interviews. All questions were asked in reference to the past year and the order of the questions was altered from the original. Additionally, in APMS, participants who scored two or above on the SCOFF questionnaire were asked whether their feelings about food interfere with their ability to work, meet personal responsibilities, and/or enjoy a social life. The SCOFF has been shown to have high sensitivity (84–100 %) and specificity (87–94 %) across validation studies utilising data from primary care patients [18].

#### Body mass index

Body mass index (BMI) was calculated using participants’ self-reported height and weight collected during the phase 1 interview. BMI is a measure of body size and may be used to indicate whether or not an individual’s weight may be considered healthy in proportion to their height. BMI was available for 3927 female participants. Participants with missing BMI values were excluded from the analyses, as we did not deem it appropriate to estimate this. Cases with BMI lower than 15 or higher than 50 were excluded as outliers (*n* = 298), as these most likely represented typing errors, rather than genuine values. This data trimming is common in the literature and was previously used in a similar study by McBride et al. [13].

#### PTSD, depression and suicide attempts

PTSD, depression, and suicide attempts were assessed, in addition to a range of other common mental disorders, using the structured Clinical Interview Schedule Revised (CIS-R) [19]. The presence of any suicide attempt across the lifespan was assessed with a single item.

### Analytic plan

Cases with over 20 % of missing data were removed prior to the analysis, resulting in the effective sample size of 3845 participants. The remaining missing values were estimated in Mplus 7.3 [20], which was used for the LCA and the regressions. All analyses were weighted to ensure representativeness to the English population. Initially, a LCA was conducted to determine the number of disordered eating classes based on participants’ responses to the five categorical SCOFF indicators and the continuous BMI indicator. Six models that included 1 through to 6 classes were specified and estimated using the default robust maximum likelihood estimator. The relative fit of the models was assessed using Akaike’s Information Criterion (AIC), Bayesian Information Criterion (BIC), and the sample size-adjusted BIC (SSABIC). Lower values of these indices indicate a better-fitting model. Entropy was used as a measure of the accuracy of classification with values closer to unity indicating high accuracy. In addition, the Lo-Mendell-Rubin adjusted likelihood ratio test (LMR) was used to directly compare models with different numbers of classes. Significant values (*p* < .05) suggest that a given model fits better than another with one fewer class. Each model was estimated with three different sets of starting values to ensure the best solution.

The second phase of the analysis utilised multinomial logistic regression. Each individual was assigned into their most likely latent class and childhood sexual (CSA) and physical abuse (CPA) were used as predictors of latent class membership whilst controlling for age, marital status, ethnicity and educational level.

In the final phase of the analysis, the mental health outcomes of depression, PTSD and suicide attempts were entered into the multinomial logistic regression model as outcomes of disordered eating profiles. CSA, CPA, age, ethnicity, educational level and marital status were all controlled for.

## Results

Table 1 shows the demographic characteristics of the sample. Based on a SCOFF score of two or above, the past year self-reported prevalence of potential EDs was 9.2 %. This number decreased to 2.6 % when only those cases were considered who scored two or above on SCOFF and for whom feelings about food interfered with their ability to work, meet personal responsibilities, and/or enjoy a social life. The fit statistics for the six LCA models are presented in Table 2. The five-class model was considered optimal; it had the lowest BIC value, a good entropy value and the LMR test suggested that it was better than the 4- and 6-class models. The 3-class model was also acceptable based on the LMR test, as it was better than the 2- and 4-class models, however the 3-class model’s AIC, BIC, and SSABIC values were slightly higher than those of the 5-class model.

**Table 2:** Fit indices for the LCA of eating disorders

LCA model	AIC	BIC	SSABIC	Entropy	LMR
Class 1	34,014.676	34,058.458	34,036.215	N/A	N/A
Class 2	32,972.872	33,060.435	33,015.950	0.811	1037.843 (*p* = 0.0000)
Class 3	32,479.648	32,610.994	32,544.265	0.825	498.595 (*p* = 0.0000)
Class 4	32,362.460	32,537.587	32,448.616	0.809	128.957 (*p* = 0.1065)
**Class 5**	**32,242.813**	**32,461.721**	**32,350.508**	**0.815**	**131.374 (** ***p*** **=** **0.0066)**
Class 6	32,213.833	32,476.523	32,343.067	0.826	42.249 (*p* = 0.2035)

*AIC* Akaike’s information criterion, *BIC* Bayesian information criterion, *SSABIC* sample size-adjusted BIC, *LMR* adjusted Lo-Mendell-Rubin likelihood ratio test

The optimal model is shown in bold

**Fig. 1:**
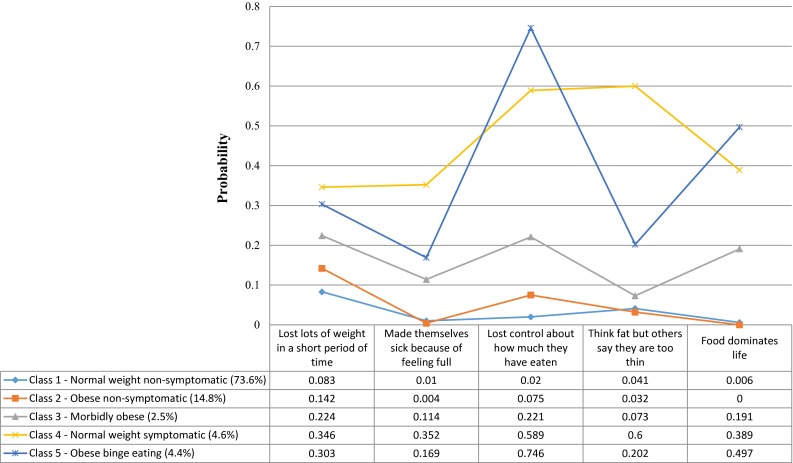
Profile plot and probabilities from the LCA

Figure 1 shows the profile plot of the 5-class model with the associated probabilities of endorsing the five SCOFF items. The size of each class was determined based on the most likely class membership for each individual. Class 1 was the largest class consisting of 73.6 % (*n* = 2832) of the sample and can be characterised by normal weight (BMI: *M* = 23.63, SE = 0.14) and low endorsement of all SCOFF indicators. This class was labelled “Normal weight non-symptomatic”. Class 2 consisted of 14.8 % (*n* = 570) of the sample and it also had a relatively low endorsement of all SCOFF indicators. The average BMI for this class was in the obese range (*M* = 31.73, SE = 0.51) and it was labelled “Obese non-symptomatic”. Class 3 was the smallest class consisting of 2.5 % (*n* = 94) of the sample. This class had a moderate endorsement of all SCOFF indicators and had BMI in the morbidly obese range (*M* = 41.55, SE = 0.81). We labelled this class as “Morbidly obese”. Class 4 consisted of 4.6 % (*n* = 179) of the sample and had an average BMI in the normal range (*M* = 22.93, SE = 0.67). Members of this class had a very high probability of endorsing the items ‘Lost control about how much they have eaten’ and ‘Think fat but others say they are too thin’. The endorsement of the remaining SCOFF items was also high in this class, which was labelled “Normal weight symptomatic”. 4.4 % of the sample (*n* = 170) belonged to class 5, and these individuals had a very high probability of endorsing the item ‘Lost control about how much they have eaten’ and a high probability of endorsing the item ‘Food dominates life’. The endorsement of the remaining items was moderate and the average BMI was in the obese range (*M* = 30.77, SE = 0.93). The class was labelled “Obese binge eating”.

Results of the multinomial logistic regression with the trauma and demographic variables as predictors of latent classes are presented in Table 3. Trauma was found to be a significant predictor of the disordered eating profiles. Individuals who have experienced CSA were almost twice as likely to be in latent class 5 (obese binge eating) and over three times as likely to be in latent class 4 (normal weight symptomatic) or latent class 3 (morbidly obese) than in the reference group (normal weight non-symptomatic). The odds for being in latent class 2 (obese non-symptomatic) for those who experienced CSA, were increased but not significant. With regard to CPA, the survivors were over four times more likely to be in the ‘obese binge-eating’ class, over three times more likely to be in the ‘Morbidly obese’ class and over twice as likely to be in the ‘obese non-symptomatic’ class, than in the reference group. CPA survivors also had increased odds of being in the ‘normal weight symptomatic’ class, however these were not significant.

**Table 3:** Odds ratios (95 % confidence intervals) for demographic and trauma variables as predictors of disordered eating latent class membership

Predictors	Class 2 (obese non-symptomatic)	Class 3 (morbidly obese)	Class 4 (normal weight symptomatic)	Class 5 (obese binge eating)
Trauma type				
Childhood sexual abuse	1.13 (0.87–1.47)	3.07 (1.87–5.03)***	3.34 (2.16–5.17)***	1.98 (1.33–2.93)**
Childhood physical abuse	2.07 (1.27–3.37)**	3.49 (1.66–7.33)**	1.59 (0.71–3.57)	4.41 (2.46–7.91)***
Age (55 and above = reference)				
16–34	0.55 (0.38–0.78)**	0.77 (0.29–2.07)	5.47 (2.60–11.54)***	3.53 (1.97–6.35)***
35–54	0.92 (0.72–1.17)	2.44 (1.33–4.48)**	3.40 (1.69–6.85)**	3.04 (1.86–4.99)***
Ethnicity (white = reference)				
Black	2.95 (1.73–5.01)***	2.11 (0.81–5.48)	0.56 (0.15–2.15)	1.16 (0.46–2.94)
Asian	0.67 (0.27–1.64)	0.65 (0.09–4.88)	0.62 (0.16–2.33)	1.37 (0.48–3.90)
Mixed other	0.78 (0.34–1.79)	2.76 (0.97–7.88)	0.64 (0.18–2.29)	0.64 (0.18–2.31)
Education (no qualifications = reference)				
Higher level qualification	0.55 (0.41–0.75)***	0.18 (0.08–0.42)***	1.29 (0.67–2.50)	1.18 (0.70–2.01)
A level	0.95 (0.66–1.37)	0.62 (0.29–1.31)	2.11 (1.05–4.24)*	1.70 (0.91–3.19)
GCSE or equivalent	1.06 (0.81–1.39)	0.76 (0.41–1.40)	1.92 (1.05–3.52)*	1.53 (0.90–2.61)
Foreign/other	0.89 (0.54–1.47)	0.73 (0.16–3.29)	0.96 (0.19–4.79)	0.99 (0.25–4.00)
Marital status (married/cohabiting = reference)				
Single	0.64 (0.45–0.91)*	0.79 (0.39–1.61)	1.78 (1.10–2.87)*	0.73 (0.44–1.22)
Previously married/cohabiting	1.00 (0.80–1.25)	0.97 (0.56–1.66)	1.19 (0.71–1.98)	1.43 (0.93–2.19)

Reference group = class 1 (normal weight non-symptomatic)

* *p* < .05

** *p* < .01

*** *p* < .001

The analysis of demographic variables revealed that being between the age of 16–54 was associated with increased odds for being in the ‘obese binge eating’ class. Being single, between 16 and 54 years old and having GCSEs or A levels as the highest educational qualification, was associated with significantly elevated odds for being in the ‘normal weight symptomatic’ class. With regard to the ‘morbidly obese’ class, individuals in the 35–54 age category were more likely to be in this class and individuals with a higher level educational qualification were less likely to be in this class than in the reference group. Individuals who were single, between 16 and 34 years of age and those with a higher level educational qualification were less likely to be in the ‘obese non-symptomatic’ class and those with a black racial background were more likely to be in that class compared to the reference group.

Table 4 shows the results of the multinomial logistic regression of the ED profiles, trauma types and demographic variables as predictors of suicide attempts, PTSD and depression. Individuals in the ‘obese binge eating’ class were over twice as likely to have attempted suicide and to have PTSD, and over four times as likely to be suffering from depression when compared to the reference group (normal weight non-symptomatic). Members of the ‘normal weight symptomatic’ class were over twice as likely to have attempted suicide, and more than three times as likely to be suffering from PTSD and depression, compared to the reference group. The ‘morbidly obese’ class was over three times as likely as the reference group to be suffering from depression. The odds ratios for attempted suicide and PTSD in the ‘morbidly obese’ group were elevated, but not significant. With regard to the ‘obese non-symptomatic’ class, no significant associations with any of the mental health outcomes were found. As for the trauma types, survivors of CSA had significantly increased odds for having attempted suicide and for suffering from PTSD and depression. CPA significantly predicted suicide attempts and depression.

**Table 4:** Odds ratios (95 % confidence intervals) for disordered eating profiles, trauma types and demographics as predictors of mental health outcomes

Predictors	Suicide attempts	PTSD	Depression
Eating disorder profiles (class 1 normal weight non-symptomatic = reference)			
Class 2 (obese non-symptomatic)	1.36 (0.89–2.07)	1.09 (0.59–2.02)	1.37 (0.78–2.40)
Class 3 (morbidly obese)	1.80 (0.83–3.91)	2.43 (0.94–6.28)	3.46 (1.66–7.18)**
Class 4 (normal weight symptomatic)	2.58 (1.45–4.60)**	3.18 (1.57–6.42)**	3.52 (1.72–7.20)**
Class 5 (obese binge eating)	2.83 (1.65–4.84)***	2.64 (1.30–5.38)**	4.38 (2.44–7.86)***
Trauma type			
Childhood sexual abuse	3.41 (2.41–4.82)***	3.09 (1.92–4.98)***	1.94 (1.26–2.99)**
Childhood physical abuse	3.57 (2.20–5.82)***	1.67 (0.80–3.49)	2.65 (1.44–4.89)**
Age			
16–34	1.81 (1.04–3.15)*	2.52 (1.25–5.09)*	0.84 (0.43–1.62)
35–54	1.67 (1.09–2.55)*	3.19 (1.81–5.63)***	2.07 (1.24–3.45)**
Ethnicity (white = reference)			
Black	0.29 (0.08–1.02)	0.89 (0.28–2.84)	0.55 (0.15–2.00)
Asian	0.24 (0.08–0.76)*	0.49 (0.15–1.66)	2.36 (0.69–8.04)
Mixed other	0.46 (0.15–1.42)	0.44 (0.10–1.98)	1.17 (0.40–3.45)
Education (no qualifications = reference)			
Higher level qualification	0.51 (0.31–0.83)**	0.64 (0.36–1.16)	0.60 (0.35–1.04)
A level	0.37 (0.20–0.70)**	0.35 (0.17–0.70)**	0.81 (0.40–1.63)
GCSE or equivalent	0.76 (0.48–1.22)	0.51 (0.28–0.93)*	0.89 (0.54–1.48)
Foreign/other	1.29 (0.61–2.70)	0.32 (0.06–1.85)	0.55 (0.14–2.11)
Marital status (married/cohabiting = reference)			
Single	1.22 (0.75–1.97)	1.78 (0.97–3.29)	2.35 (1.42–3.87)**
Previously married/cohabiting	1.87 (1.31–2.68)**	2.27 (1.40–3.67)**	2.74 (1.77–4.24)***

Reference group = not endorsing suicide attempts, PTSD, or depression

* *p* < .05

** *p* < .01

*** *p* < .001

The analysis of demographic variables revealed that being between the ages of 16–54 and being previously married/cohabiting increased the odds for attempted suicide, whereas having Asian ethnic background and having obtained A levels or a higher level educational qualification significantly decreased the odds. With regard to PTSD, being previously married/cohabiting and being between 16 and 54 years old increased the odds of suffering from the disorder, and having either GCSEs or A levels as the highest educational qualification decreased the odds. Finally, being in the 35–54 age group and being either single or previously married/cohabiting was associated with increased odds for suffering from depression.

## Discussion

The aim of this study was to identify profiles of disordered eating in a nationally representative sample of females aged 16 and above and to examine the associations of these with childhood sexual and physical maltreatment and specific mental health outcomes. The past year prevalence of potential EDs in our sample was 2.6 % (based on a SCOFF score of two or above and having feelings about food that interfere with one’s ability to work, meet personal responsibilities, and/or enjoy a social life), which is relatively high compared to other population-based studies [21]. However, this could be attributed to the fact that the SCOFF questionnaire is not a diagnostic tool, but rather a screening measure designed to simply alert to the possibility of an ED.

The LCA of five SCOFF indicators and the self-reported BMI yielded five classes. Class 1 (‘normal weight non-symptomatic’) was the largest class consisting of individuals whose BMI was in the normal range and who, on average, did not show elevated symptoms of EDs. Class 2 (‘obese non-symptomatic) individuals were obese, but could likewise be considered non-symptomatic in terms of EDs. With regard to the additional variables entered in the model, it was found that those reporting CPA were more likely to be in the ‘obese non-symptomatic’ class relative to the reference group, which is in line with the previous literature supporting the importance of childhood trauma as a factor in obesity [22]. These individuals, however, were no more or less likely to have attempted suicide or to be suffering from depression or PTSD than the reference group. Interestingly, having black racial background (as opposed to white) increased the likelihood of being in the ‘obese non-symptomatic’ class. The higher probability of black (compared to white) individuals being obese without elevated symptoms of EDs could be explained in terms of the differences in cultural ideals between the two groups. Research suggests that the black culture promotes a heavier body type [23] and black women tend to be more satisfied with their body and less likely to adopt media messages equating beauty with thinness [24].

The remaining three latent classes were our disordered eating classes. Class 3 (‘morbidly obese’) had the highest average BMI and showed moderate endorsement of all SCOFF indicators. Patterns of disordered eating are highly prevalent in bariatric surgery patients [25] as well as primary care patients with moderate to severe obesity [26] and can include EDs as well as sub-clinical disturbances [25]. Survivors of CPA and/or CSA were over three times more likely to be in the ‘morbidly obese’ class as opposed to the reference class. These results support those from other cross-sectional [27] and longitudinal studies [28]. Compared to the reference group, the ‘morbidly obese’ individuals were also at an elevated risk for depression, which again is in line with the existing literature, especially studies conducted with females and those with severe obesity [29].

Class 4 (‘normal weight symptomatic’) was our most symptomatic class. Compared to the other four latent classes, individuals in this group were at least three times more likely to think that they are fat despite others telling them they are too thin, which is one of the diagnostic criteria for AN in DSM-5 [2]. This disorder can be accompanied by binge eating and purging, which were likewise highly endorsed in this group. Binge eating is also found in cases of BN [2] and since the SCOFF questionnaire is not a diagnostic tool, it is possible that cases of BN were also included in this class. Membership in this group was significantly predicted by CSA; a finding which supports previous research, and especially studies focusing on binge eating behaviours [30]. Carter, Bewell, Blackmore and Woodside [31] found that CSA is frequently reported by individuals seeking treatment for AN, and Sanci et al. [10] reported that the incidence of the bulimic syndrome was higher in those females who had experienced CSA than those who did not. Individuals in the ‘normal weight symptomatic’ class were also at an increased risk for associated depression, PTSD, and suicide attempts. Significant associations of depression with AN or BN have been reported in previous studies [32] and the presence of PTSD should not be surprising, as it is a common outcome of CSA [33]. Moreover, it has also been found that outpatients with AN, especially those with the purging type, are at an elevated risk for attempting suicide [34]. Increased risk has also been reported for BN cases [35]. Noteworthy is also the finding that participants who reported being single and who were younger were more likely to be in the ‘normal weight symptomatic’ group than the reference group, conforming to the typical profiles of AN and BN patients.

Class 5 (‘obese binge eating’) was another highly symptomatic class with BMI in the obese range and the highest probabilities of endorsing the items ‘lost control about how much they have eaten’ and ‘food dominates life’. This pattern resembles, to some extent, the DSM-5 diagnosis of BED [2] and fits in with the studies reporting strong associations between BED and food addiction [36]. With regard to childhood maltreatment, individuals with experiences of CSA and CPA were more likely to belong to the ‘obese binge eating’ class than reference class. In previous studies, both CSA and CPA have been linked to obesity [22], binge eating behaviours [37], and also food addiction [38]. With regard to mental health outcomes, the ‘obese binge eating’ class had the highest odds for attempted suicide and developing depression. Odds ratios for PTSD were likewise significantly elevated. Previous studies have reported significant positive associations between binge eating, obesity, and depressive symptoms [39], binge eating and PTSD [14], binge eating and suicide attempts [40], and there is also some evidence for the positive association between obesity and attempted suicide, specifically in females [41].

Taken together, these results suggest that childhood trauma is significantly associated with disordered eating, which in turn is significantly associated with depression, PTSD, and suicide attempts in a representative sample of English females. The three disordered eating classes were the most affected ones in terms of childhood trauma and adverse mental health outcomes. The ‘morbidly obese’ class was the least affected disordered eating class in terms of mental health psychopathology, which could be explained by the moderate endorsement of the SCOFF items in this class. Indeed, previous studies have shown that severity of mental health psychopathology increases with increasing severity of EDs [42]. ‘Normal weight symptomatic’ and ‘obese binge eating’ classes were the most affected ones, supporting previous literature on mental health psychopathology in individuals with EDs [7]. Our results are also consistent with previous American and Australian twin cohort studies which reported the highest rates of mental health psychopathology in the classes characterised by the highest severity of disordered eating [43, 44].

The results of this study should be considered in light of several limitations. First, the SCOFF questionnaire is not a clinical measure, but rather a screening tool and as such it does not enquire about all features of AN, BN, or the other recognized EDs. It is, therefore, not possible to say whether all individuals categorized into one of our three disordered eating classes would actually be diagnosed with ED. This matter is further complicated by the fact that BMI in APMS was based on self-reported weight and height. Although research suggests that self-reported weight and height are reasonably accurate and reliable [45], obese individuals and females are more likely to underestimate their BMI [46] and underweight females are more likely to overestimate it [47], which could explain, to some extent, the normal weight of individuals in our ‘normal weight symptomatic’ class. It is also possible that cases with severe AN and other ED diagnoses were not included in the APMS as this was a household survey and did not include individuals who were hospitalised at the time of the interviews.

The second limitation of our study is that the APMS questions used to assess early adverse experiences are not the gold standard in the field. CSA and CPA were assessed using three and one questions, respectively, and these could not have covered the whole range of experiences that would qualify as CSA or CPA. Moreover, CSA included one item which asked if the participants have experienced sexual talk. It could be argued that including non-contact abuse in the definition could lead to higher prevalence estimates; however, Pereda and colleagues [48] found no difference in the prevalence rates between the narrow definition, which included only contact CSA and the broad definition, which included non-contact CSA.

Finally, the study is limited by its cross-sectional nature, which prevents us from drawing definitive conclusions regarding the temporal order of the childhood maltreatment, ED onset, and mental health outcomes. However, the existing prospective longitudinal studies suggest that childhood trauma is a significant predictor of later onset EDs [49] and disordered eating can predict the onset of depression [50]. In addition, APMS lacks data on the age of onset of the individual mental health problems as well as the age at which the child maltreatment occurred, which further complicates our conclusions about the time sequence of the events in our sample. Longitudinal studies are needed to resolve this issue.

Despite these limitations, the current study was the first to analyse the profiles of disordered eating and associated trauma experiences and psychopathology in a nationally representative, female-only sample of adults living in private households in England. Three groups of individuals with disordered eating patterns were identified and a history of CSA or CPA increased the likelihood of females being grouped into one of these classes relative to the non-symptomatic reference class. The classes with higher symptomatology of EDs were at increased risk for depression, PTSD, and suicide attempts. These results highlight the need to screen for EDs in individuals with a history of childhood maltreatment presenting with symptoms of depression, PTSD and/or suicidal ideation.
